# Haemodynamics in the mouse aortic arch computed from MRI-derived velocities at the aortic root

**DOI:** 10.1098/rsif.2012.0295

**Published:** 2012-07-04

**Authors:** Mark A. Van Doormaal, Asimina Kazakidi, Marzena Wylezinska, Anthony Hunt, Jordi L. Tremoleda, Andrea Protti, Yvette Bohraus, Willy Gsell, Peter D. Weinberg, C. Ross Ethier

**Affiliations:** 1 Department of Bioengineering, MRC Clinical Sciences Centre, Imperial College London; 2 Biological Imaging Centre, MRC Clinical Sciences Centre, Imperial College London

**Keywords:** mouse, shear stress, blood flow, atherosclerosis

## Abstract

Mice are widely used to investigate atherogenesis, which is known to be influenced by stresses related to blood flow. However, numerical characterization of the haemodynamic environment in the commonly studied aortic arch has hitherto been based on idealizations of inflow into the aorta. Our purpose in this work was to numerically characterize the haemodynamic environment in the mouse aortic arch using measured inflow velocities, and to relate the resulting shear stress patterns to known locations of high- and low-lesion prevalence. Blood flow velocities were measured in the aortic root of C57/BL6 mice using phase-contrast MRI. Arterial geometries were obtained by micro-CT of corrosion casts. These data were used to compute blood flow and wall shear stress (WSS) patterns in the arch. WSS profiles computed using realistic and idealized aortic root velocities differed significantly. An unexpected finding was that average WSS in the high-lesion-probability region on the inner wall was actually higher than the WSS in the low-probability region on the outer wall. Future studies of mouse aortic arch haemodynamics should avoid the use of idealized inflow velocity profiles. Lesion formation does not seem to uniquely associate with low or oscillating WSS in this segment, suggesting that other factors may also play a role in lesion localization.

## 1. Introduction

Mouse models of chronic diseases such as atherosclerosis are valuable tools for understanding human pathology. Indeed, the use of genetically modified strains has been a major breakthrough in investigations of the biological pathways involved in atherogenesis [1,2]. Lesions are readily induced in the larger arteries of susceptible strains, and these vessels are also accessible for imaging; the aortic arch has received particular attention [1–4].

It is well known that atherogenesis is influenced by haemodynamic factors, the most prominent of which is wall shear stress (WSS). Several studies [5–10] have examined WSS patterns in the mouse aortic arch by numerically simulating blood flow, for which both the accurate arch geometry and inflow boundary conditions (blood velocity profiles at the aortic root) must be known. Previous studies have used idealized (e.g. parabolic or Wormersley) velocity profiles at the root. Using a more physiologically realistic profile could have a significant impact on computed WSS patterns. More specifically, flow in a curved vessel such as the aortic arch typically results in a velocity profile that is skewed towards the outer curvature, reducing WSS on the inner curvature [11], although this effect is reversed near the inlet of a curved tube under certain circumstances [12]; a skewed inlet velocity profile could intensify or weaken this effect. The use of physiologically accurate profiles is therefore important to our understanding of the relationship between atherogenesis and biomechanical factors.

Our goals in this work were to measure temporally and spatially resolved mouse-specific blood velocity profiles at the aortic root and to determine their effect on aortic arch haemodynamics. We used MRI to measure velocities, corrosion casts to determine aortic arch geometries and numerical modelling to compute WSSs. We found that using realistic aortic root velocity profiles had an impact on computed aortic arch haemodynamics, and that our blood flow simulations predicted a surprising similarity between the haemodynamic environment in regions known to differ in their predilection for the formation of atherosclerotic-like lesions.

## 2. Material and methods

### 2.1. Animals

Animals were housed in a temperature- (21 ± 2°C), humidity- (50 ± 10%), and light- (non-inverted 12 L : 12 D cycle) controlled environment and given ad libitum access to food and water. Ten adult C57BL6 male mice (Harlan, UK) were imaged, of which five animals had datasets of sufficient quality for subsequent use (table 1).

**Table 1.** Physiological data for mice MR scanned to quantify the blood velocity profile at the aortic root. Heart rate, respiratory rate and body temperature were measured at least once for every gradient echo image during the MR scan (20–60 recordings per mouse); the reported values are mean ± sample s.d.

date of scan	age (weeks)	body mass (grams)	heart rate (bpm)	respiration rate (breaths per minute)	temperature (°C)
070909AM	7.5	22.9	509 ± 21	49 ± 14	36.9 ± 0.2
070909PM	7.5	22.7	534 ± 30	31 ± 6	36.9 ± 0.6
210909AM	8	22.2	497 ± 9	24 ± 5	36.6 ± 0.3
210909PM	8	22.9	470 ± 19	37 ± 14	36.7 ± 0.3
230909	8	23.3	588 ± 13	41 ± 14	36.6 ± 0.0

Anaesthesia was induced with 4 per cent isoflurane in 100 per cent oxygen and maintained with approximately 1.5 per cent isoflurane in 1.5 l min^–1^ O_2_ via a facemask, while the mouse lay in the supine position on a platform. Subdermal ECG needle electrodes and a respiratory cuff (SA Instruments, Stony Brook, NY, USA) were placed for monitoring heart and respiratory rate, respectively. Body temperature was monitored via a rectal probe and maintained at 37°C (36.7 ± 0.2°C) using a heated-air system (SA Instruments, Stony Brook, NY, USA). For the remainder of this work, all mice will be referenced according to the date of the MRI scan (e.g. 210909AM).

### 2.2. Magnetic resonance imaging

**Figure 1.**
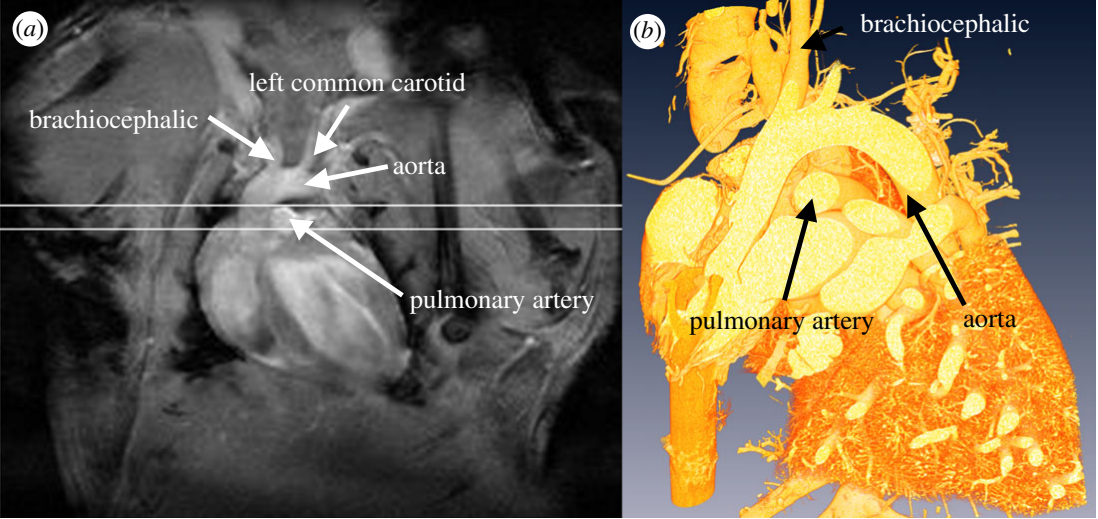
Anatomical MRI scan used for planning, showing the slice used for MR velocimetry at (*a*) the aortic root in white and (*b*) a corresponding cross-sectional slice through the three-dimensional rendered CT scan of the post mortem cast. Both images from mouse 210909AM.

The MRI slice used for velocimetry was centred on the pulmonary artery (figure 1). Details of imaging protocols are given in the electronic supplementary material, supplementary methods. For one-dimensional blood flow velocity encoding, two sets of gradient echo fast low angle shot (FLASH) images were acquired with all imaging parameters kept constant, except for the first moment of the flow encoding gradient. Flow was measured separately along three orthogonal directions: through-plane, and in-plane along phase and frequency encoding directions. Based on preliminary images, the waveforms of the through-plane flow encoding gradient were calculated to fulfil the requirement that a peak velocity (VENC) of 140 cm s^−1^ corresponded to a phase shift of π. VENC for the smaller in-plane (secondary) velocities were iteratively decreased to 40 cm s^−1^, except for mouse 070909AM which was scanned with an in-plane VENC of 50 cm s^−1^. Other imaging parameters were TE = 1.50 ms; TR = 18 ms; four acquired images averaged at each time point; 1 mm slice thickness; FOV = 30 × 30 mm; and a 256 × 256 pixel matrix, giving an in plane resolution of 0.117 mm.

Data acquisition was performed with dual cardiac and respiratory gating while preserving steady-state condition of longitudinal magnetization. The respiratory rate during acquisition was 36 ± 10 breaths per minute (mean ± s.d.) and the average heart rate was 520 ± 45 bpm for the five mice used in this study. Measurements of blood flow were taken every 6 ms during the cardiac cycle at time points from 0 to 48 ms after the onset of systole, as defined by the R wave and a 4 µs trigger delay.

### 2.3. Processing of the MRI velocity data

**Figure 2.**
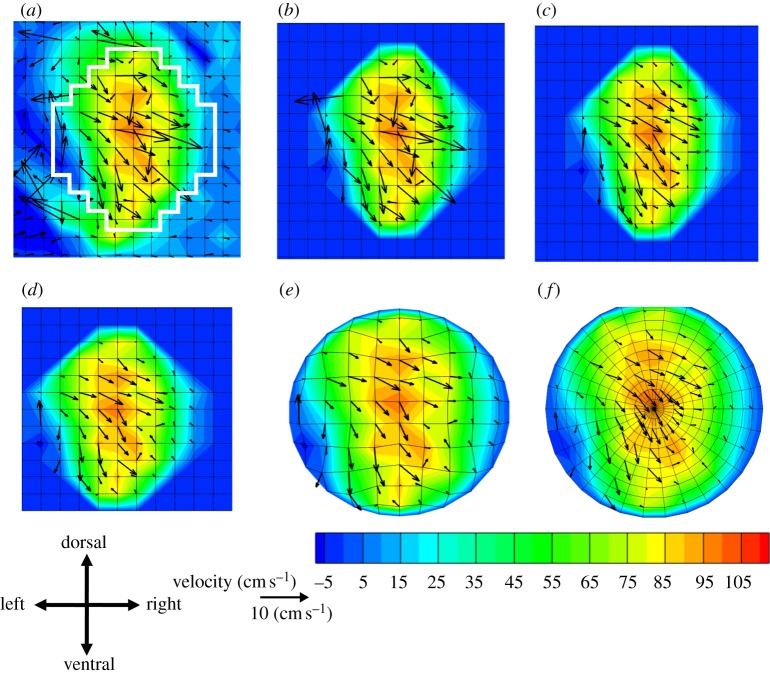
MRI data at different stages of processing. (*a*) Unwrapped, offset data with vessel mask shown in white, (*b*) masked data, (*c*) corrected for spurious vectors, (*d*) corrected for vessel movement, (*e*) warped to circle and (*f*) interpolated to mesh with constant radial and azimuthal spacing. Colour contours of the through-plane velocity and in-plane velocity vectors are shown.

Several post-processing steps were required (figure 2). First, a mask was created that identified voxels in the lumen of the ascending aorta. It was determined manually using a combination of the anatomical and velocity data. The size and shape of the mask changed over the cardiac cycle as the aorta moved. Second, the in-plane velocity fields contained vectors that differed substantially from their neighbours and were thus likely erroneous. Such spurious vectors are also a feature of particle image velocimetry datasets, and methods have been developed to detect and correct them. We used the dynamic mean operator [13] to detect spurious vectors and an inverse distance interpolation of nearest, non-spurious neighbours to correct them.

Blood velocity was measured in the magnet's reference frame but the subsequent blood flow modelling and inter-mouse comparison required velocities expressed relative to the moving vessel wall. In order to compensate for vessel movement, vessel wall velocity was calculated using the no-slip condition (fluid velocity next to the wall must equal the wall velocity). ‘Border nodes’ that were inside the artery but had a neighbour outside the artery were identified from the ascending aorta mask, and the average velocity over all border nodes was taken as vessel wall velocity at each time step. To compensate for vessel movement, this velocity was subtracted from the measured blood velocity.

To allow inter-mouse comparisons, datasets were mapped to a common circular domain and rotated so that the dorsoventral axis of each mouse was in a common orientation. A consistent circular domain was created by warping the aortic root to fit to a circular domain without changing any velocity values. The dorsoventral axis was defined by determining the geometric centres of the spine and sternum from the MRI magnitude images. Further details of post processing are given in the electronic supplementary material, supplementary methods.

### 2.4. Micro-CT post processing

Aortic geometries were obtained by micro-CT scanning of corrosion casts obtained post-mortem. Scans were segmented to isolate the geometry of interest and create a three-dimensional surface model that could be used as the basis for a computational mesh for blood flow modelling (see the electronic supplementary material, supplemental methods). The cast from 210909PM showed artefacts and was not used in computational blood flow modelling, although the MR blood velocity data were unaffected and were included when computing average velocities.

### 2.5. Blood flow modelling

Blood flow and WSS patterns were computed numerically as described in the electronic supplementary material, supplementary methods. On the basis of the four mice with good casts and MR velocimetry data from five mice, seven flow simulations were completed as follows. One simulation was performed to compute unsteady aortic blood flow in each of the four mice with good casts, using mouse-specific inlet velocities determined by MRI and the corresponding cast geometry. Three additional unsteady simulations were performed using the cast geometry of mouse 210909AM. In the first, only the through-plane MRI velocities were specified at the aortic root, the in-plane components being set to zero. This simulation explored the importance of the in-plane velocity components on the WSS field. The second used a uniform aortic root velocity profile in the through-plane direction and no in-plane velocity components, with the flow rate matched to the full MRI measurements. This simulation was compared with the previous one to explore the effects of using idealized rather than realistic through-plane inlet velocities. In the third, mouse-averaged, unsteady MRI-determined velocities were applied in order to determine how a realistic but non-subject-specific set of velocity conditions compares to subject-specific aortic conditions.

## 3. Results

**Figure 3.**
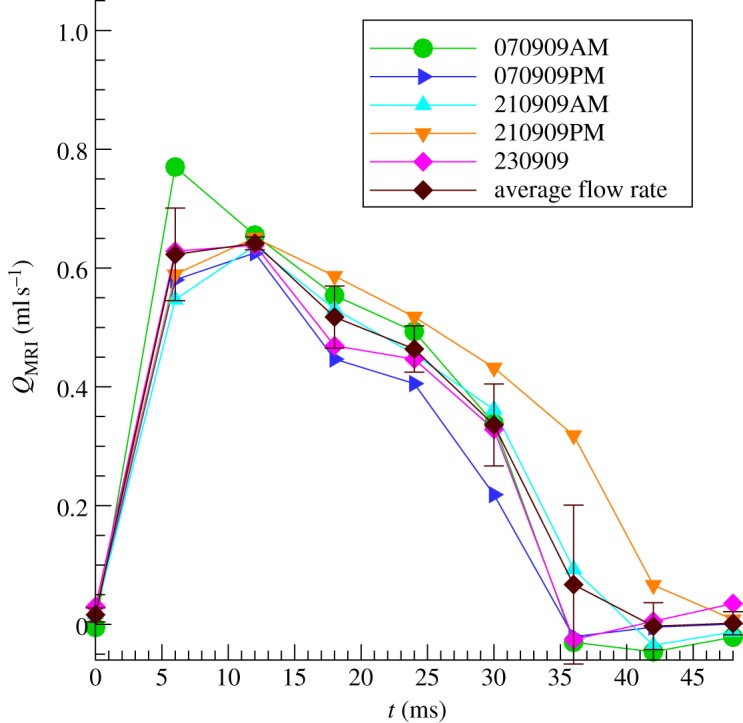
Flow rate versus time for five mice, as well as the average. The flow rate was determined by integrating the through-plane velocity over the vessel area (given by the ascending aorta mask) at each time-step.

A plot of flow rate as a function of time for the five mice with good MRI scans is shown in figure 3. There was good agreement among mice with respect to the peak flow rate and the general shape of the flow waveform. Mouse 210909PM had notably more prolonged flow than the other mice, and also the lowest heart rate (table 1).

**Figure 4.**
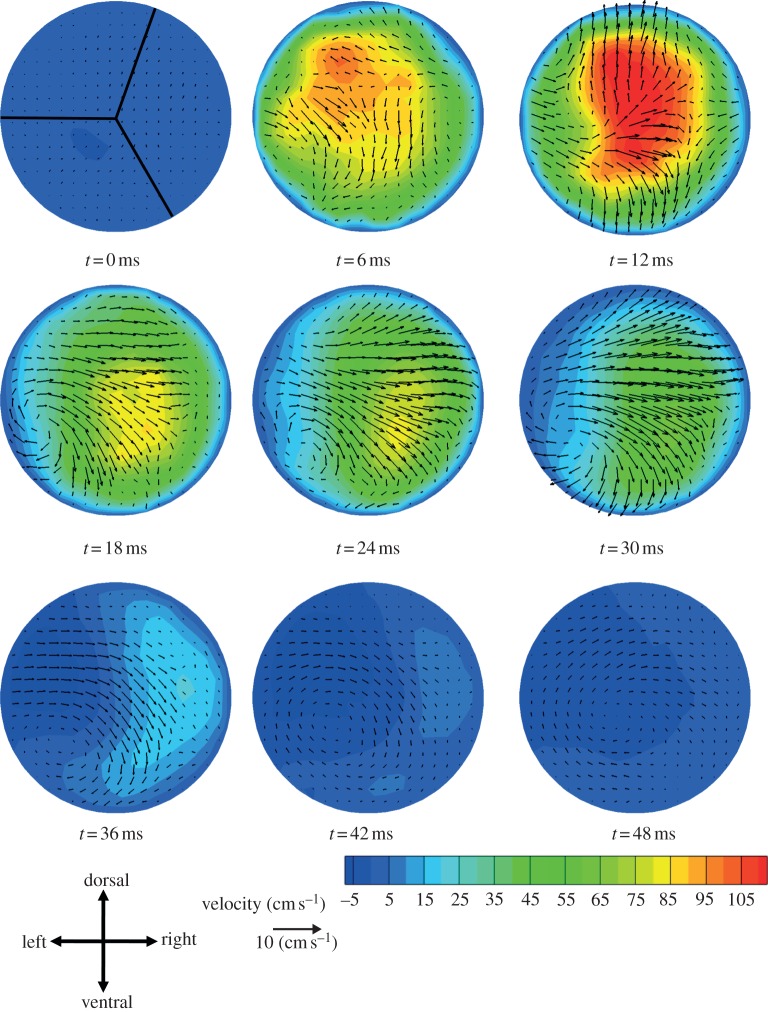
Aortic root blood velocity averaged across mice as described in the text. At *t* = 0 ms the approximate location of the valve leaflets in a fully closed position is shown. Contours show through-plane velocity and vectors show in-plane velocity.

**Figure 5.**
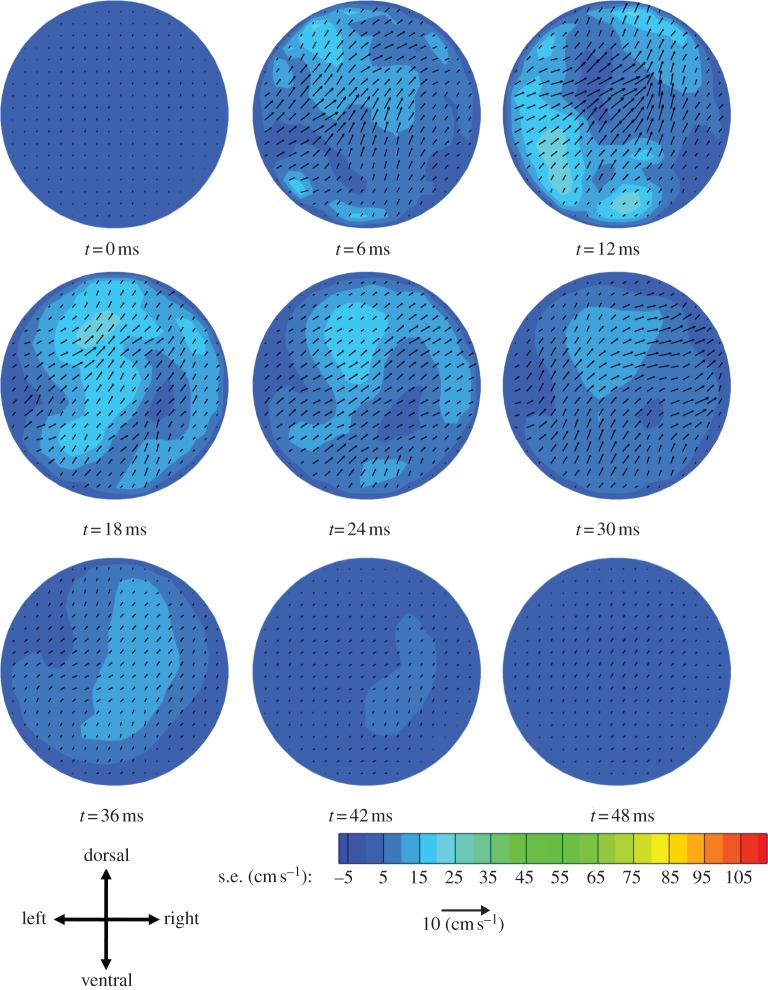
Contours and vectors representing s.e. of the mean for the mouse-averaged velocities shown in figure 4. Contours and vectors represent the s.e. of the mean for through-plane and in-plane velocities, respectively. Because the s.e. is always positive, the vectors point in the positive *x*- and *y*-directions. The contours and vectors are shown using the same scale as figure 4 to allow comparison between the s.e. and the mean values.

Aortic root blood velocity data for these five mice showed complex flows with some common features. In order to better understand blood flow features at the aortic root that were common among mice, we computed a mouse-averaged velocity field and the standard error of the mean for this velocity field (figures 4 and 5). (Individual data are given in the electronic supplementary material, supplementary results.) The velocity patterns showed several differences to analogous measurements in humans [14]. The through-plane standard error (contours in figure 5) was relatively low for the first six time points (up until *t* = 30 ms); for *t* > 30 ms, the error was of the same order as the velocity itself. The standard error in the in-plane directions (vectors in figure 5) was of the same order as the in-plane velocity at all time points.

At *t* = 0 ms, the mouse-averaged velocity field exhibited only small velocities. At *t* = 6 ms, there was through-plane acceleration of the blood while the in-plane flow was noticeably disorganized, with few structures being evident. At *t* = 12 ms, close to peak systole, the through-plane velocities reached a maximum near the centre of the vessel with the highest-speed fluid zone extending slightly in the dorsal and ventral directions. The in-plane flow displayed more organized behaviour, although distinct structures were difficult to discern. At 18 ms there was a deceleration of the through-plane flow compared with the 12 ms pattern, and the highest-speed fluid zone moved towards one side of the vessel. This behaviour was maintained in the through-plane flow pattern at 24 ms. At both 18 and 24 ms, the in-plane data displayed clockwise rotating vortices that slowly dissipated over the duration of the cardiac cycle. At 30 and 36 ms, the deceleration continued, as did the movement of the highest-speed fluid zone towards the right-ventral direction. Additionally, the flow on the left side of the vessel was reduced greatly and even reversed slightly. At 42–48 ms, there was little through-plane flow and the in-plane flow was reduced to weak clockwise vortices.

### 3.1. The impact of inlet flow conditions on aortic arch wall shear stress patterns

**Figure 6.**
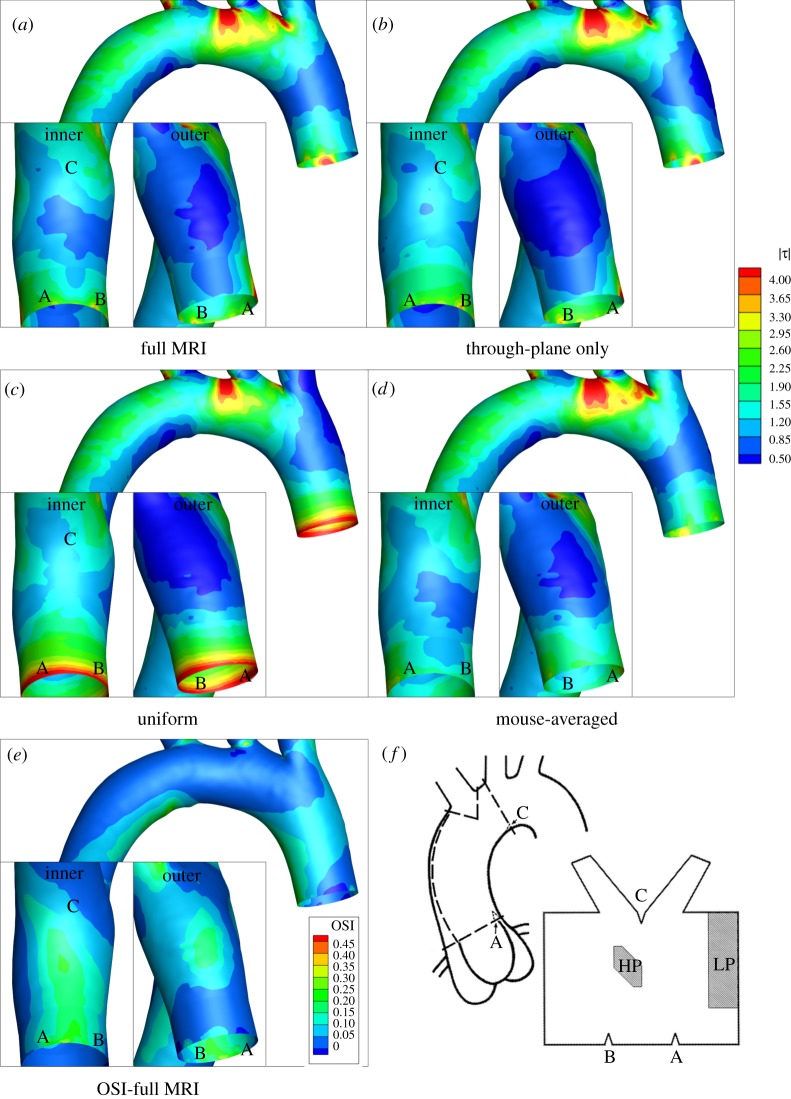
(*a*–*d*) Normalized wall shear stresses (WSSs) and (*e*) oscillatory shear index (OSI) patterns in the aortic arch of mouse 210909AM for various velocity boundary conditions. Anatomical markers A, B and C are shown in WSS and OSI panels as well as in relation to areas of high-probability (HP) and low-probability (LP) of atherosclerotic lesion formation (*f*, from Iiyama *et al.* [4]). Each panel shows the ventral side (main pictures), inner curvature between the aortic root and first branch (left insets), and outer curvature between the aortic root and the first branch (right insets). All WSSs have been normalized by the value that would exist in a long, straight arterial segment having the diameter of the aortic root with the same inflow conditions, which for this mouse is 60 dynes cm^−2^.

As described already, four different unsteady aortic root velocity profiles were used to investigate the effect of inlet boundary conditions on WSS patterns in the aortic arch of one mouse. (Instantaneous streamlines at two time points in this mouse are given in the electronic supplementary material, supplementary results.) Figure 6 shows that the global WSS distribution was not greatly affected by the inlet velocity profiles, especially at the more distal locations of the arch, with the exception of localized changes around the second branch ostium. On the other hand, there were appreciable effects on detailed patterns of WSS, notably on the critical inner (figure 6, left insets) and outer wall (figure 6, right insets) of the proximal section, where inhomogeneities in lesion prevalence have been demonstrated. Specifically, normalized WSS values on the inner wall (figure 6, left insets) were greater when using the through-plane only inflow velocities (figure 6*b*) than when using the full MRI velocities (figure 6*a*); they were even greater when using a flat (uniform) velocity profile at the aortic root (figure 6*c*). The opposite was true on the outer wall near the inlet (figure 6, right insets), where the WSS values decreased when going from full MRI to through-plane only to flat velocity conditions.

Figure 6 also demonstrates that using realistic but not subject-specific velocity conditions (mouse averaged, figure 6*d*) resulted in WSS patterns that were very similar to those obtained by using subject-specific conditions.

Oscillatory shear index (OSI) is a measure of how much the shear stress reverses direction over the cardiac cycle. It was defined by He & Ku [15] as
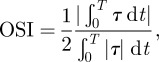
where *τ* is the WSS vector and *T* is the duration of the cardiac cycle. OSI values on the inner and outer wall were higher than elsewhere (including the lateral walls), with a marginally higher value over a larger area on the inner wall than the outer (figure 6*e*). Thus, both the inner and outer walls of the arch before the first branch were subjected to low levels of WSS and broadly similar levels of OSI.

For reference, the locations of high (HP) and low (LP) probability of lesion formation, as defined by Iiyama *et al.* [4], are shown in figure 6*f*. The location of markers and the corresponding regions of high- and low-probability of lesion development are approximate, given inter-mouse variation in aortic arch geometry and the schematic nature of the diagram used to locate the HP and LP zones.

**Figure 7.**
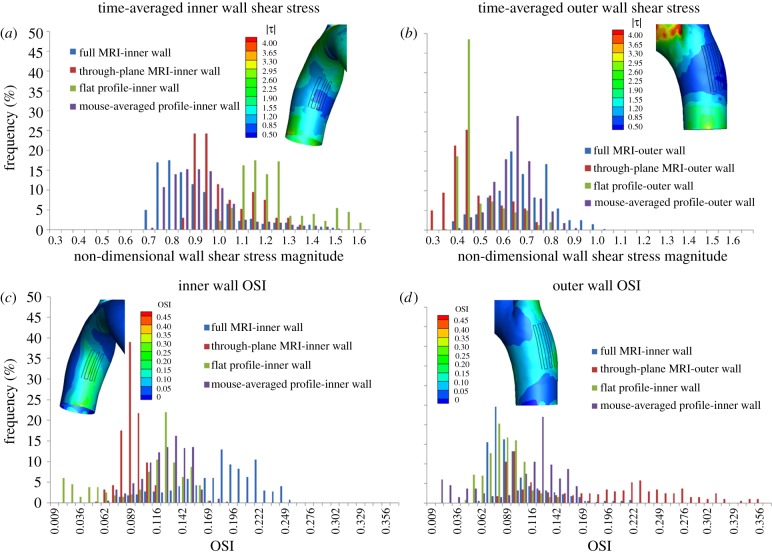
Histograms of normalized time-averaged WSS values on patches placed on (*a*) the inner curvature and (*b*) outer curvature of mouse 210909AM for different inlet velocity conditions, and of OSI for the same mouse on (*c*) the inner and (*d*) outer curvatures. The inset in each panel shows the corresponding contours of WSS and OSI, respectively, for the case of the full MRI inlet conditions and with the patch region visible as an undulating black line. The area of the inner curvature patch is slightly (22%) smaller than the area of the outer curvature patch. Each patch was composed of a series of line segments, with four hundred equally spaced nodes along the line. WSS magnitude was normalized as described in figure 4.

Shear stresses were further quantified within ‘patches’ on the luminal surface of the inner and outer walls (figure 7) that were chosen to coincide with the HP and LP areas identified by Iiyama *et al.* [4]. Both patches were made slightly larger than these areas in order to ensure overlap with them, because it was difficult to precisely map the areas onto our geometry. The inner wall patch was approximately 50 per cent larger than the HP area of Iiyama *et al.* and the outer wall patch was approximately 25 per cent larger than the LP area.

A histogram of the WSS levels occurring within each patch (figure 7) confirmed that WSSs on the inner wall increased as inlet boundary conditions were changed from full MRI-measured velocities to through-plane only MRI measurements to the flat inlet profile, with the opposite holding true on the outer wall. Figure 7 also confirms that the WSS on the outer patch was lower in magnitude than the WSS on the inner patch. This was quantified by calculating average values: shear stress was 0.882 (53 dynes cm^−2^; range 0.68–1.46) for the inner wall patch and 0.664 (40 dynes cm^−2^; range 0.36–1.00) for the outer wall patch, using the full MRI inlet conditions. Finally, figure 7 also confirms quantitatively that using the mouse-averaged inflow velocity data gives results that closely followed the full MRI data.

Similar histograms of OSI (figure 7) show that the dependence of OSI on inlet conditions is greater than the dependence of WSS on these conditions. We also observe that there is lower OSI in the outer curvature patch compared with the inner curvature patch, an observation that seems to support the idea that high OSI (i.e. low and oscillating WSS) promotes lesions. However, further examination from a slightly different orientation (figure 6*e*) shows that the relatively low OSI patch seen in figure 7*d* is immediately adjacent to a region of high OSI on the outer curvature, which is not a site of lesion formation. This emphasizes the fact that our data do not show a *consistent* association between low and/or oscillatory WSS and sites known to have a predilection for lesion formation.

## 4. Discussion

The region of the mouse ascending aorta with the highest probability of developing lipid deposition is the inner wall (lesser curvature) of the distal segment, before the first branch, while the area with the lowest probability is the outer wall (greater curvature) of the same segment [4] (figure 6*f*). This variation has been used to investigate the possible dependence of lesion prevalence on mechanical stress. Endothelial cells within the high probability (HP) region are less elongated and more randomly aligned than those in the low probability (LP) region, consistent with an expected difference in shear stresses [16]. Previous studies [5–10] have systematically mapped the pattern of WSS in these regions. However, these studies have assumed idealized aortic root blood velocity profiles. This work investigated the effect of using more realistic inflow velocity conditions on computed shear stress patterns. Through-plane and in-plane velocities were obtained from phase contrast MR images and high-resolution anatomical information was obtained from micro-CT of vascular corrosion casts. This approach is subject to certain limitations, which should not affect the overall conclusions [17].

WSS patterns were generally insensitive to inflow conditions at locations distal to the first branch with the exception of localized changes around the second branch ostium. However, in regions between the inlet and the first branch, the choice of inlet boundary conditions affected the level of WSS on both the inner and outer wall. When using a flat inlet velocity profile, the WSS was under-estimated on the outer wall and over-estimated on the inner wall, compared with using the full MRI inlet velocity boundary conditions. The absence of in-plane velocity components contributed to this effect; excluding them again caused an under-estimation of WSS on the outer wall and over-estimation on the inner wall. In fact, the presence of swirling (in plane) velocities at the aortic root appears to reduce spatial and temporal variations in WSS, consistent with previous studies [18,19] where swirl induced by out-of-plane curvature reduced spatial variations in WSS.

These results demonstrate the importance of using realistic inflow data when computing shear stresses in the mouse aortic arch. Such data are not easy to obtain. It may therefore also be important that the non-subject specific (average mouse) inlet velocity boundary conditions contained coherent through-plane and in-plane flow structures and resulted in a very similar WSS pattern to those computed using the full (mouse-specific) boundary conditions. This may justify the use of our mouse-average inlet velocity field (see the electronic supplementary material) as a reasonable approximation of the actual velocity field in future studies. However, it should be borne in mind that velocities are likely to depend on factors such as age, weight, sex and strain as well as the anaesthetic used.

As in previous studies [5–10], low WSS was found on the inner wall (normalized shear stresses less than one), coinciding with the HP region. The presence of this patch of WSS was, as expected, attributable to the development of counter-rotating vortices, much like the Dean vortices forming in curved tubes under steady flow conditions. Another feature of the WSS patterns was the presence of an area of low values on the outer wall of the arch, between the aortic root and the first branch. By analysing the average WSS in patches on the inner and outer walls, we unexpectedly found that the time-averaged WSS in the LP region was actually lower than in the HP region (figures 6 and 7).

The low WSS zone on the outer wall resulted from a recirculation zone on the upstream wall of the first branch. Recirculation occurred at this location (and at the equivalent sites in the other branches) at peak systole because the flow failed to change direction to enter the branch and became detached from the wall (see the electronic supplementary material, supplementary results). The recirculation zones were themselves characterized by relatively slow-moving fluid near the wall and hence low WSS. Additionally, the recirculation zone provided a barrier to flow entering the first branch along the outer wall of the arch. The fluid decelerated and was forced outwards in the dorsal and ventral directions away from the central plane of the arch. The deceleration and secondary flow also caused low WSS.

This feature of the WSS pattern was generally consistent across different mice when using subject-specific geometry and inlet velocity data. However, individual variation in geometry could disrupt it: the patch of low shear was not seen in one mouse (070909PM) that had tighter aortic arch curvature than the other mice, an arch that started to curve closer to the aortic root and a more distal position of the first branch. These features caused earlier and stronger formation of the Dean-type secondary flow, skewing the highest velocity toward the outer wall and hence causing higher levels of WSS there.

Other authors have not explicitly remarked on the presence of a similar low WSS region on the outer curvature, although because this region has not previously been the focus of attention, many existing studies do not show clear images of the WSS distribution in this area. However, the study of Trachet *et al*. [20] does demonstrate a remarkably similar low WSS region (see their fig. 2 for young adult mice), and is entirely consistent with our findings. In reviewing studies that do show cycle-averaged WSS distributions on the outer curvature, it became clear that the existence of a low shear region on the outer curvature can be suppressed by sharp arch curvature and a more distally located brachiocephalic ostium, which is consistent with our findings among our cohort of mice and may explain why this region was not observed and/or commented on by others. Another factor affecting this low WSS zone may be the flow split at the brachiocephalic trunk. In a recent study, Trachet *et al*. [21] carefully measured volumetric flow rate from the major branches of the aorta, observing more flow entering the brachiocephalic than we specified (22.5% versus our value of 15.3%). Using this higher value would in fact make the separation effect that we observe more pronounced, suggesting that our assumed flow splits may have underestimated the extent of the low WSS zone in our mice.

The coincidence of low WSS and *high* lesion probability on the inner wall is consistent with the low WSS theory of atherogenesis [22]. On the other hand, the coincidence of low WSS and *low* lesion probability suggests that factors in addition to low and oscillatory WSS could be important in atherogenesis. The apparent importance of other localizing factors in atherogenesis could be explained by strain-dependent differences in aortic arch geometries. Different strains can have different aortic arch geometries [8], and arch geometry can affect WSS patterns on the outer wall as evidenced by mouse 070909PM. The LDLR^−/−^ mice used by Iiyama *et al.* when mapping lesion prevalence were of mixed 129-C57BL/6 background while the strain for our MRI velocity measurements was C57BL/6; Zhu *et al.* [8] found differences in arch geometry between C57BL/6 and 129/SvEv mice. We must also keep in mind the possibility that the haemodynamic environment could differ between the wild-type (WT) mice used in this study and the knockout (KO) mice used in lesion mapping studies. Potential sources for such differences include arch geometry, the magnitude and waveform of cardiac output, blood viscosity and arterial stiffness. These issues have been studied by others. Zhu *et al.* [8] did not observe large differences in arch geometry between WT and KO mice. One study reported a higher cardiac output in older KO mice versus WT [23], while another [24] reported the opposite for younger mice, and flow rates in the KO mice varied by a factor of two; despite this wide range, there is a clear predilection for lesions to form on the inner, as opposed to the outer, curvature of the aortic arch in Apo-E^−/−^ and LDLR^−/−^ mice [25], indicating that disease localization in KO mice is not particularly sensitive to cardiac output. Finally, KO mice were reported to have an 11 per cent lower haematocrit and a 13 per cent higher pulse-wave velocity versus WT mice [23], but these are expected to have only modest effects on arch haemodynamics, where flow patterns are dominated by curvature, branch outflow rates and inlet velocity profile. We therefore believe that our haemodynamic results are valid for KO mice, but further confirmatory haemodynamic studies in KO mice are warranted.

Another possibility, recently suggested by Zhou *et al*. [26], is that retrograde flow rather than low shear stress is the localizing factor leading to lesion development. This suggestion was based on Doppler ultrasound measurements that showed late systolic/early diastolic retrograde flow on the inner but not the outer curvature of the ascending aorta, and by the pro-atherogenic effect of experimentally increasing retrograde flow. It is consistent with the earlier numerical results of Suo *et al*. [6], which indicated a greater change in flow direction during the cardiac cycle at the inner wall than the outer wall. Our own results (figure 6) also show greater changes in direction (characterized by the OSI) on the inner than those on the outer wall. However, both these regions had much higher OSI values than other parts of the ascending aorta. Hence, the lowest OSI regions were not those associated with the lowest lesion prevalence, and areas associated with the lowest lesion prevalence had moderately high values of OSI. The apparent discrepancy with the results of Suo *et al*. and Zhuo *et al*. may derive from the fact that reverse flow was characterized at only a few points in the ascending aorta in these earlier studies; our maps show significant variation of OSI over short distances (figure 6). The discrepancy may also reflect the limited resolution of the Doppler measurements, the use of a flat inlet profile in the earlier numerical study, or other technical differences such as the compensation for effects of anaesthesia in the present study (see the electronic supplementary material, supplementary methods).

Alternatively, low WSS and oscillatory flow may be important, but the measures of WSS level and oscillations that we used, namely cycle-averaged WSS and OSI, respectively, may not be the indicators most closely associated with disease propensity, even though they are widely used in the literature. For example, atherogenesis might depend on the amount of time in the cardiac cycle that endothelial cells are exposed to WSS below a certain threshold rather than on the cycle-averaged WSS level, or on fluctuations in flow direction other than the complete changes in sign captured by the OSI.

Of course, a further possibility is that atherogenesis is not *exclusively* due to low and/or oscillating shear stress, however parametrized. For example, atherogenesis may depend on the mass transfer of blood-borne molecules (e.g. LDL and NO) between blood and endothelium, which is usually, but not inevitably, related to WSS [17,27]. In rabbit models, there is evidence for a dependence of lesions on wall strain [28] and, as here, for association with variables in addition to low shear [29]. It is relevant in this context that wall strain is likely to vary between the inner and outer walls of the aortic arch, and that it can influence endothelial cell elongation [30].

Scaling laws dictate that many aspects of mouse haemodynamics differ from those in people [31]. Indeed, the mechanisms driving atherogenesis may differ between mouse and human arteries. Nonetheless, we suggest that further study of the role in atherogenesis of mechanobiological factors in addition to low/oscillatory WSS is warranted.

## References

[1] Lichtman A. H. Clinton S. K. Iiyama K. Connelly P. W. Libby P. Cybulsky M. I. 1999 Hyperlipidemia and atherosclerotic lesion development in LDL receptor-deficient mice fed defined semipurified diets with and without cholate. Arterioscler. Thromb. Vasc. Biol. 19, 1938–1944 10.1161/01.ATV.19.8.1938 (doi:10.1161/01.ATV.19.8.1938) 10446074

[2] Nakashima Y. Plump A. S. Raines E. W. Breslow J. L. Ross R. 1994 ApoE-deficient mice develop lesions of all phases of atherosclerosis throughout the arterial tree. Arterioscler. Thromb. 14, 133–140 10.1161/01.ATV.14.1.133 (doi:10.1161/01.ATV.14.1.133) 8274468

[3] Daugherty A. 2002 Mouse models of atherosclerosis. Am. J. Med. Sci. 323, 3–10 10.1097/00000441-200201000-00002 (doi:10.1097/00000441-200201000-00002) 11814139

[4] Iiyama K. Hajra L. Iiyama M. Li H. DiChiara M. Medoff B. Cybulsky M. 1999 Patterns of vascular cell adhesion molecule-1 and intercellular adhesion molecule-1 expression in rabbit and mouse atherosclerotic lesions and at sites predisposed to lesion formation. Circ. Res. 85, 199–207 10.1161/01.RES.85.2.199 (doi:10.1161/01.RES.85.2.199) 10417402

[5] Feintuch A. 2007 Hemodynamics in the mouse aortic arch as assessed by MRI, ultrasound, and numerical modeling. Am. J. Physiol. Heart Circ. Physiol. 292, H884–H892 10.1152/ajpheart.00796.2006 (doi:10.1152/ajpheart.00796.2006) 17012350

[6] Suo J. Ferrara D. Sorescu D. Guldberg R. Taylor R. Giddens D. 2007 Hemodynamic shear stresses in mouse aortas: implications for atherogenesis. Arterioscler. Thromb. Vasc. Biol. 27, 346–351 10.1161/01.ATV.0000253492.45717.46 (doi:10.1161/01.ATV.0000253492.45717.46) 17122449

[7] Huo Y. Guo X. Kassab G. 2008 The flow field along the entire length of mouse aorta and primary branches. Ann. Biomed. Eng. 36, 685–699 10.1007/s10439-008-9473-4 (doi:10.1007/s10439-008-9473-4) 18299987

[8] Zhu H. Zhang J. Shih J. Lopez-Bertoni F. Hagaman J. Maeda N. Friedman M. 2009 Differences in aortic arch geometry, hemodynamics, and plaque patterns between C57BL/6 and 129/SvEv mice. J. Biomech. Eng. 131, 121005 20524728 10.1115/1.4000168 PMC3047446

[9] Trachet B. Swillens A. Van Loo D. Casteleyn C. De Paepe A. Loeys B. Segers P. 2009 The influence of aortic dimensions on calculated wall shear stress in the mouse aortic arch. Comp. Methods Biomech. Biomed. Eng. 12, 491–499 10.1080/10255840802695445 (doi:10.1080/10255840802695445) 19221921

[10] Vandeghinste B. Trachet B. Renard M. Casteleyn C. Staelens S. Loeys B. Segers P. Vandenberghe S. 2011 Replacing vascular corrosion casting by *in vivo* micro-CT imaging for building 3D cardiovascular models in mice. Mol. Imaging Biol. 13, 78–86 10.1007/s11307-010-0335-8 (doi:10.1007/s11307-010-0335-8) 20449667

[11] Dean W. R. 1927 Note on the motion of fluid in a curved pipe. Phil. Mag. 20, 208–223

[12] Caro C. G. Pedley T. J. Schroter R. C. Seed W. A. 1978 The mechanics of the circulation. Oxford, UK: Oxford University Press

[13] Raffel M. Willert C. Kompenhans J. 2002 Particle image velocimetry: a practical guide (experimental fluid mechanics). Berlin, Germany: Springer

[14] Jin S. Oshinski J. Giddens D. 2003 Effects of wall motion and compliance on flow patterns in the ascending aorta. J. Biomech. Eng. 125, 347–354 10.1115/1.1574332 (doi:10.1115/1.1574332) 12929239

[15] He X. Ku D. N. 1996 Pulsatile flow in the human left coronary artery bifurcation: average conditions. J. Biomech. Eng. 118, 74 10.1115/1.2795948 (doi:10.1115/1.2795948) 8833077

[16] Hajra L. Evans A. Chen M. Hyduk S. Collins T. Cybulsky M. 2009 The NF-kB signal transduction pathway in aortic endothelial cells is primed for activation in regions predisposed to atherosclerotic lesion formation. Proc. Natl Acad. Sci. USA 97, 9052–9057 10.1073/pnas.97.16.9052 (doi:10.1073/pnas.97.16.9052) 10922059 PMC16820

[17] Van Doormaal M. 2010 Numerical modelling of cardiovascular flows: from in vitro systems to animal models. PhD thesis, Imperial College London, London, UK

[18] Caro C. G. Doorly D. J. Tarnawski M. Scott K. T. Long Q. Dumoulin C. L. 1996 Non-planar curvature and branching of arteries and non-planar-type flow. Proc. R. Soc. Lond. A 452, 185–197 10.1098/rspa.1996.0011 (doi:10.1098/rspa.1996.0011)

[19] Sherwin S. J. Shah O. Doorly D. J. Peiro J. Papaharilaou Y. Watkins N. Caro C. G. Dumoulin C. L. 2000 The influence of out-of-plane geometry on the flow within a distal end- to-side anastomosis. J. Biomech. Eng. 122, 86 10.1115/1.429630 (doi:10.1115/1.429630) 10790834

[20] Trachet B. Swillens A. Van Loo D. Casteleyn C. De Paepe A. Loeys B. Segers P. 2009 The influence of aortic dimensions on calculated wall shear stress in the mouse aortic arch. Comput. Methods Biomech. Biomed. Eng. 12, 491–499 10.1080/10255840802695445 (doi:10.1080/10255840802695445) 19221921

[21] Trachet B. Bols J. De Santis G. Vandenberghe S. Loeys B. Segers P. 2011 The impact of simplified boundary conditions and aortic arch inclusion on CFD simulations in the mouse aorta: a comparison with mouse-specific reference data. J. Biomech. Eng. 133, 121 006 10.1115/1.4005479 (doi:10.1115/1.4005479) 22206423

[22] Caro C. G. Fitz-Gerald J. M. Schroter R. C. 1971 Atheroma and arterial wall shear. Observation, correlation and proposal of a shear dependent mass transfer mechanism for atherogenesis. Proc. R. Soc. Lond. B 177, 109–133 10.1098/rspb.1971.0019 (doi:10.1098/rspb.1971.0019) 4396262

[23] Hartley C. J. 2000 Hemodynamic changes in apolipoprotein E-knockout mice. Am. J. Physiol. Heart Circ. Physiol. 279, H2326–H2334 11045969 10.1152/ajpheart.2000.279.5.H2326

[24] Tomita H. Hagaman J. Friedman M. H. Maeda N. 2012 Relationship between hemodynamics and atherosclerosis in aortic arches of apolipoprotein E-null mice on 129S6/SvEvTac and C57BL/6J genetic backgrounds. Atherosclerosis 220, 78–85 10.1016/j.atherosclerosis.2011.10.020 (doi:10.1016/j.atherosclerosis.2011.10.020) 22078246 PMC3246113

[25] Maeda N. Johnson L. Kim S. Hagaman J. Friedman M. Reddick R. 2007 Anatomical differences and atherosclerosis in apolipoprotein E-deficient mice with 129/SvEv and C57BL/6 genetic backgrounds. Atherosclerosis 195, 75–82 10.1016/j.atherosclerosis.2006.12.006 (doi:10.1016/j.atherosclerosis.2006.12.006) 17275002 PMC2151972

[26] Zhou Y.-Q. Zhu S.-N. Foster S. Cybulsky M. Henkelman M. 2010 Aortic regurgitation dramatically alters the distribution of atherosclerotic lesions and enhances atherogenesis in mice. Arterioscl. Thromb. Vasc. Biol. 30, 1181–1188 10.1161/ATVBAHA.110.204198 (doi:10.1161/ATVBAHA.110.204198) 20299687

[27] Coppola G. Caro C. 2008 Oxygen mass transfer in a model three-dimensional artery. J. R. Soc. Interface 5, 1067–1075 10.1098/rsif.2007.1338 (doi:10.1098/rsif.2007.1338) 18252664 PMC2607427

[28] Thubrikar M. J. Baker J. W. Nolan S. P. 1988 Inhibition of atherosclerosis associated with reduction of arterial intramural stress in rabbits. Arterioscl. Thromb. Vasc. Biol. 8, 410–420 10.1161/01.ATV.8.4.410 (doi:10.1161/01.ATV.8.4.410) 3395277

[29] Bond A. R. Iftikhar S. Bharath A. A. Weinberg P. D. 2011 Morphological evidence for a change in the pattern of aortic wall shear stress with age. Arterioscler. Thromb. Vasc. Biol. 31, 543–550 10.1161/ATVBAHA.110.219683 (doi:10.1161/ATVBAHA.110.219683) 21205986

[30] Zhao S. Suciu A. Ziegler T. Moore J. Burki E. Meister J.-J. Brunner H. 1995 Synergistic effects of fluid shear stress and cyclic circumferential stretch on vascular endothelial cell morphology and cytoskeleton. Arterioscl. Thromb. Vasc. Biol. 15, 1781–1786 10.1161/01.ATV.15.10.1781 (doi:10.1161/01.ATV.15.10.1781) 7583556

[31] Weinberg P. D. Ethier C. R. 2007 Twenty-fold difference in hemodynamic wall shear stress between murine and human aortas. J. Biomech. 40, 1594–1598 10.1016/j.jbiomech.2006.07.020 (doi:10.1016/j.jbiomech.2006.07.020) 17046000

